# Meditation and complexity: a review and synthesis of evidence

**DOI:** 10.1093/nc/niaf013

**Published:** 2025-05-28

**Authors:** Daniel A Atad, Pedro A.M Mediano, Fernando E Rosas, Aviva Berkovich-Ohana

**Affiliations:** Edmond Safra Brain Research Center, Faculty of Education, University of Haifa, Haifa 3103301, Israel; The Integrated Brain and Behavior Research Center (IBBRC), University of Haifa, Haifa 3103301, Israel; Faculty of Education, Department of Counseling and Human Development, University of Haifa, Haifa 3103301, Israel; Department of Computing, Imperial College London, 180 Queen’s Gate, South Kensington, London SW7 2RH, United Kingdom; Department of Psychology, University of Cambridge, Downing Site, Downing Place, London, Cambridge CB2 3EB, United Kingdom; Sussex AI and Centre for Consciousness Science, Department of Informatics, University of Sussex, Brighton BN19RH, London, United Kingdom; Centre for Psychedelic Research, Department of Brain Science, Imperial College London, 180 Queen’s Gate, South Kensington, London SW7 2RH, United Kingdom; Centre for Eudaimonia and Human Flourishing, University of Oxford, London, Oxford OX39BX, United Kingdom; Centre for Complexity Science, Imperial College London, 180 Queen’s Gate, South Kensington, London SW7 2RH, United Kingdom; Edmond Safra Brain Research Center, Faculty of Education, University of Haifa, Haifa 3103301, Israel; The Integrated Brain and Behavior Research Center (IBBRC), University of Haifa, Haifa 3103301, Israel; Faculty of Education, Department of Counseling and Human Development, University of Haifa, Haifa 3103301, Israel; Faculty of Education, Department of Learning and Instructional Sciences, University of Haifa, Haifa 3103301, Israel

**Keywords:** meditation, consciousness, complexity, entropy, fractal dimension, predictive processing, neuroimaging, literature review

## Abstract

Recent years have seen growing interest in the use of metrics inspired by complexity science for the study of consciousness. Work in this field has shown remarkable results in discerning conscious from unconscious states, and in characterizing states of altered conscious experience following psychedelic intake as involving enhanced complexity. Here, we study the relationship between complexity and a different kind of altered state of consciousness: meditation. We provide a scoping review of the growing literature studying the complexity of neural activity in meditation, disentangling different families of measures, short-term (state) from long-term (trait) effects, and meditation styles. Beyond families of measures used, our review uncovers a convergence toward identifying higher complexity during the meditative state when compared to waking rest or mind-wandering and decreased baseline complexity as a trait following regular meditation practice. In doing so, this review contributes to guide current debates and provides a framework for understanding the complexity of neural activity in meditation, while suggesting practical guidelines for future research.

## Introduction

Meditation refers to contemplative practices that involve regulating both body and mind through cultivating a state of heightened awareness (Cahn and Polich 2006), and includes a wide variety of techniques and approaches (Goleman 1988). A large body of ongoing work converges on the positive effects of meditation, including enhanced emotion regulation, attentional skills, and wellbeing, as well as stress reduction (Jha et al. 2007, Chiesa and Serretti 2010, Farb et al. 2012, Zollars et al. 2019, Querstret et al. 2020, Tang et al. 2022). Meditation has also been related to alterations in the regular sense of self (Dahl et al. 2015; reviewed by Tang et al. 2015) and alleviation of the symptoms of various mental conditions (Zhihong et al. 2018, Parmentier et al. 2019, Geurts et al. 2021, Haider et al. 2021). Given these important implications, uncovering the neural correlates of meditation is a crucial challenge that, if solved, could help us improve our scientific understanding of consciousness, and the implementation of meditation-assisted psychotherapy. While early work focused on uncovering underlying mechanisms of meditation in the frequency domain mostly via electroencephalography (EEG), providing insights into the power or intensity of different frequency bands in brain activity (reviewed by Cahn and Polich 2006), there have been many efforts in recent years to uncover cortical regions and networks, examining the functional magnetic resonance imaging (fMRI) activity and connectivity patterns between brain regions and functional networks (e.g. reviewed by Fox et al. 2016, Fox and Cahn 2018, Young et al. 2018).

In addition to these more traditional approaches, recent years have seen a growing interest in the use of complexity-inspired measures in the study of consciousness, particularly in the characterization of different altered states of consciousness (ASCs) (partially reviewed in Sarasso et al. 2021, Lau et al. 2022). In general, ASCs refer to temporary states in which there is a significant qualitative shift in subjective experience, including changes in perception, thought processes, emotions, and sense of self (Tart 1972, Farthing 1992). These ASCs can be induced by various means, including meditation, psychoactive substances, hypnosis, sleep deprivation, sensory deprivation, and other interventions that alter brain function (Tart 1972, Vaitl et al. 2005). In effect, the field of complexity science (Jensen 2022) has developed widely applicable theories describing emergent phenomena generated by the interplay of many parts of a system. When applied to the highly complex system of the human brain (Sporns 2022), these theories allow a description of the informational richness and self-organization seen in brain activity. As these approaches offer complementary insights into brain functioning, beyond the classical approaches of frequency or network activity and connectivity, they should be combined to gain a more comprehensive understanding of the brain’s meditation-induced intricate dynamics.

To date, complexity research of meditation lacks an explicit organizing framework, which should include theory, empirical comparison, and accepted guidelines for conducting research. To fill this gap, here we present a comprehensive review on the existent work relating meditation and complexity, providing an overview of conceptual issues and a review of the existing empirical studies. In the following, we first describe the notion of complexity and entropy of neural activity, and their use in consciousness studies. Then, we elaborate on the definition and typology of meditation, and lay out theoretical considerations on complexity and meditation, before presenting a scoping review of the current literature on the subject and an analysis of the results of this review following our proposed framework. We conclude by discussing our main findings and offering suggestions for future research in this growing field.

## Consciousness and complexity

### Metrics of complexity in neuroscience

Complexity science is a scientific discipline that aims to describe systems in which relatively simple components collectively give rise to emerging system-wide behavior (Mitchell 2009). As noted by the philosopher Paul Cilliers: “a complex system cannot be reduced to a collection of its basic constituents, not because the system is not constituted by them, but because too much of the relational information gets lost in the process” (Cilliers 1998, p. 10). The human brain is one of such complex systems, where the intricate interactions between billions of nerve cells give rise to sophisticated processes involving cognition and consciousness (Turkheimer et al. 2021). In neuroscience, complexity science has provided important conceptual and computational tools to advance our understanding of brain function. These include brain networks that describe the topology of interactions between brain regions (Sporns 2011), metastability and dynamical phase transitions that characterize sudden shifts in modes of activity (Kelso 1995), criticality that distinguishes a balance between rigidity and disorder (Cocchi et al. 2017, O’Byrne and Jerbi 2022), and the integration-differentiation coexistence as a key enabler of high brain functions (Tononi et al. 1998). Furthermore, as subsequently elaborated, the development and increasing use of complexity measures for characterizing resting-state neural activity has proven to be an important tool in the study of consciousness (Sarasso et al. 2021).

Measures inspired by principles of complexity science have been found to have the capability for discerning—to some degree—changes in consciousness, presenting an opportunity for empirical convergence beyond classical approaches (Sarasso et al. 2021). Complexity measures have been applied to “spontaneous” brain activity recorded during different conscious states (e.g. Burioka et al. 2005, Schartner et al. 2015, 2017), and also to evoked neural activity following external stimulation [e.g. via transcranial magnetic stimulation (Casali et al. 2013)]. As all of the reviewed meditation studies applied complexity measures to spontaneous recordings, we will focus on this approach in our review.

Overall, empirical studies show a general trend of increase in the complexity of brain dynamics in relation to an increase in level of consciousness and/or felt richness of conscious experience—for example, differentiating between sleep stages (Burioka et al. 2005) and indexing depth of anesthesia (Zhang et al. 2001, Liang et al. 2015). Typically, states of lower conscious level, such as anesthesia and non-rapid eye movement (NREM) sleep, score lower on complexity measures than wakefulness (Burioka et al. 2005, Schartner et al. 2015). In turn, states of altered phenomenology of conscious experience including rich conscious content, such as the psychedelic state, have been systematically shown to exhibit higher complexity than normal wakefulness (Farnes et al. 2020, Lebedev et al. 2016, Li and Mashour 2019, Mediano et al. 2024, Ruffini et al. 2023; M. M. Schartner et al. 2017, Timmermann et al. 2019, Timmermann et al. 2023, Varley et al. 2020, Viol et al. 2016, for overviews see Girn et al. 2023, McCulloch et al. 2022). Importantly, the level and richness of consciousness are distinct yet highly interrelated concepts, which are actively debated (Overgaard and Overgaard 2010, Bayne et al. 2016). Grossly, conscious level refers to the degree of wakefulness or arousal—how conscious one is, while the richness of conscious content refers to its depth and detail (Ji et al. 2024). While higher levels usually support richer content, this is not always the case (Bayne et al. 2016).

While during the early days of complexity science researchers strived to find a unique and all-encompassing signature of “complexity,” there is a growing consensus that there are multiple facets of complexity (Mitchell 2009, Lau et al. 2022) and that distinguishing and differentiating them is an important contribution of complexity science. Hence, when talking about complexity it is crucial to specify what type of complexity one is focusing on. Sarasso et al. (2021) offer a provisional taxonomy of strategies to approach complexity in neuroscience: topological (spatial), temporal, and a combination of the two. When estimating complexity through the topological strategy, typically topological properties of a network of interdependencies are extracted from time series data, and the complexity of this topology is then captured by measures of network science (i.e. modularity, small-worldness, etc.). This strategy can be implemented on networks built from measures such as effective or functional connectivity, and is best applied using measurement techniques with good spatial resolution such as magnetoencephalography (MEG) or functional magnetic resonance imaging (fMRI). In the temporal strategy, complexity is estimated based on the size of the repertoire of patterns generated by the temporal dynamics of neural activity. This strategy can be implemented via measures such as Sample Entropy (SE), Permutation Entropy (PE), Lempel-Ziv complexity (LZc) and Higuchi’s Fractal Dimension (HFD), and is best applied using measurement techniques with good temporal resolution such as electroencephalography (EEG) or MEG.

While results obtained from measures utilizing temporal and spatial strategies to estimate complexity may correlate in practical scenarios, they strongly differ conceptually and algorithmically. For the sake of simplicity, the rest of this review will focus only on measures utilizing the strategy of temporal differentiation, motivated by their high empirical accuracy in capturing changes in states of consciousness (Sarasso et al. 2021).

### Entropy and fractal dimension as facets of dynamical complexity

Even within the topic of temporal complexity, there are many qualitatively different ways in which a system can be said to be complex. Here we draw a distinction between two types of complexity: *entropy* and *fractal dimension*. In essence, entropy^1^ quantifies how well one can predict the future state of a system given its past, such that more unpredictable systems are generally seen as more complex. On the other hand, fractal dimension quantifies the degree of self-similarity and scale-invariance of a system, such that a system that exhibits more intricate repeating patterns at larger scales is seen as more complex. In the following, we provide an introduction to these two facets of complexity—for further elaboration, the reader may refer to more in-depth reviews on entropy (e.g. Keshmiri 2020, Fagerholm et al. 2023) and fractal dimension (e.g. Grosu et al. 2023) in neuroscience.

#### Entropy

Entropy is an important concept in complexity science, central to thermodynamics and information theory (Thurner et al. 2017). While originally introduced to quantify heat transfer, Boltzmann and Gibbs redefined entropy as an informational property—specifically, the degree of uncertainty an observer has about the microscopic realization of a given macroscopic state (Schroeder 2000). On a separate line of inquiry, Shannon (1948) found that the same formulation of entropy was capable of characterizing various communication processes, reflecting the amount of information in a given message, or the informational capabilities of a given communication channel. E.T. Jaynes (1957) unified these seemingly disparate applications by adopting a Bayesian perspective, proposing that probabilities reflect states of knowledge of observers, and that entropy is a natural metric to quantify degrees of uncertainty whether in thermodynamics or communication.

**Figure 1:**
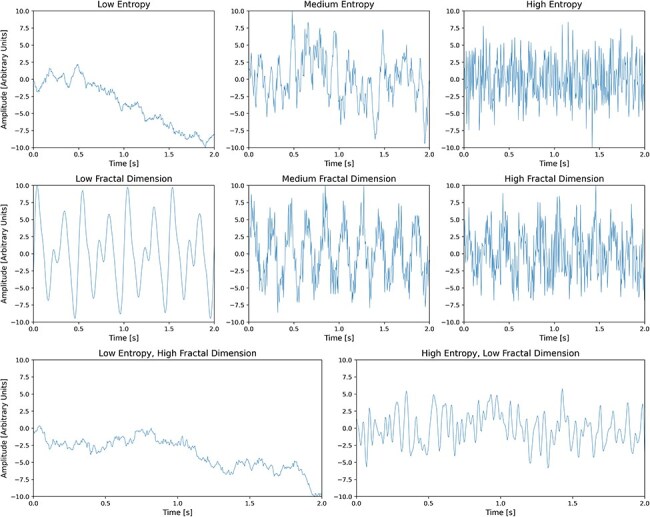
Simulated time-series sampled at 200 Hz with increasing Shannon entropy (first row) and Higuchi’s fractal dimension (second row) values. Bottom row shows two examples of time-series, where entropy and fractal dimension values differ. In all graphs “low,” “medium,” and “high” refer to the values relative to each other and should not be understood as absolute numerical values.

In the context of neural activity, entropy can be used to measure two important properties: diversity of activity and predictability (Mediano et al. 2023). On the one hand, entropy is directly related to how diverse the patterns exhibited by the neural system are, as demonstrated by the asymptotic equipartition principle.^2^ Crucially, entropy is independent of the specific patterns, only quantifying how diverse they are. On the other hand, entropy is also related to how hard it is to predict a system, providing an upper bound to the performance of an optimal predictor (Feder and Merhav 1994). Hence, entropy does not measure the performance of a specific prediction strategy, but captures the intrinsic limitations on prediction given by the statistics of the system. An illustration of the differences between time-series exhibiting low and high entropy values can be seen in Fig. 1.

The entropic brain hypothesis (Carhart-Harris et al. 2014, Carhart-Harris 2018) links entropy and consciousness, suggesting that the rich ASCs, e.g. induced by psychedelic drugs, may depend on a parallel enriching effect on the dynamics of neuronal activity, reflected in an increase of the entropy in the corresponding neuroimaging data. Conversely, ASCs involving loss of consciousness, such as anesthesia, would correspond to overly-ordered states where entropy is reduced. Crucially, the entropic brain hypothesis does not propose an identity between consciousness and entropy, but just a correlation, positing entropy as a useful marker of conscious activity. In particular, it is hypothesized (Carhart-Harris et al. 2014) that the correspondence between entropy and level of consciousness may only hold on the range of normal brain activity, but may break when entropy grows too much—e.g. hot gas is not regarded as having a very rich experience, although it has very high entropy.

#### Fractal dimension

Dimensionality refers to the number of independent variables required to describe a system or object. While dimensionality is usually associated with spatial attributes (such as the length, area, or volume), in complex systems it also encompasses dimensions in the system’s configuration space. Traditional Euclidean objects have an integer dimension (e.g. a line is 1-dimensional, a surface is 2-dimensional), yet many natural phenomena often go beyond these idealizations by exhibiting self-similarity (or self-affinity) yielding intricate patterns that repeat at different scales. Classic examples include coastlines, snowflakes, cloud formations, and stock market fluctuations (Husain et al. 2022). Mandelbrot (1967) formalized this observation by introducing the concept of “fractal dimension”, allowing for a rigorous understanding of objects exhibiting non-integer dimensions.

In this way, fractal dimension goes beyond the traditional notion of integer dimension by quantifying how a pattern fills space at different scales. A pattern that fills space more densely and exhibits more intricate details at smaller scales will have a higher fractal dimension. For dynamical systems, the fractal dimension quantifies how quickly a system fills its space of possible states as it evolves over time. Complex dynamical systems often display self-similarity and scale invariance, and their fractal dimension provides a quantitative measure of the system’s structure across different scales. A higher fractal dimension indicates a more intricate pattern within the system and thus higher complexity. In the case of neural activity, fractal dimensions offer insight into the complexity and self-organization of the underlying neural dynamics by estimating the degree of self-similarity of a signal. The fractal dimension of a time series (such as M/EEG) can be estimated either by reconstructing the multidimensional attractor that represents how neural activity evolves over time in the space of its possible states, or by directly treating the time series as a fractal pattern (Lau et al. 2022). An illustration of the differences between time-series exhibiting low and high fractal dimension values can be seen in Fig. 1.

Measures of fractal dimension also relate to the “memory” of a process (Mandelbrot 1985) defined as the amount of information about the past behavior of a system needed for optimal prediction regarding a future state. In the complexity literature, a long-memory process is said to have long-range temporal correlations (LRTC). For this reason, measures of LRTC that were found in the reviewed studies are grouped together with measures of fractal dimension, as they both provide information on the self-similarity of a stochastic process.

## Meditation and its neurological basis

In this section, we elaborate on the definition of meditation, and describe categorizations of meditation techniques. We then lay out some relevant theoretical perspectives on the neural mechanisms of meditation, drawing mainly upon predictive processing frameworks, and building on that we offer some tentative hypotheses regarding the expected changes in complexity during meditation compared to normal wakefulness.

### Meditation—a gross typology

Meditation refers to a family of contemplative practices that involve regulating both body and mind through cultivating a state of heightened awareness, which can lead to specific types of ASCs. This is typically accomplished by monitoring mental and/or physical processes, including perception, emotion, and body sensations, by employing a specific attentional set (Cahn and Polich 2006). Various cultures have developed a range of meditation techniques, resulting in many diverse practices (e.g. Goleman 1988).

Researchers have attempted to categorize these practices based on their primary aims and techniques, leading to several typologies (such as those proposed by Lutz et al. 2008, Travis and Shear 2010, Dahl et al. 2015). Despite some variations, it is generally agreed that most practices can be grouped within two broad categories: focused attention (FA) and open monitoring (OM) (Lutz et al. 2008). FA involves maintaining attention on a specific object or sensation, such as the breath, counting, or bodily sensations (examples include one-pointed awareness or visualization meditation), while OM involves cultivating a nonjudgmental and nonselective awareness of the present moment (examples include vipassana and mindfulness meditation). Additionally, some practices aim to develop emotional qualities [such as compassion and loving-kindness (LK) towards oneself and others (Lippelt et al. 2014), and some practices aim to transcend the self (ST) and experience a sense of oneness with the universe (e.g. transcendental meditation (Travis and Shear 2010) and nondual meditation (Dunne 2011)]. This categorization is helpful when attempting to group different traditions for the sake of research, yet clearly over-simplifies the practice of meditation, as many practices involve cultivating skills from different categories, even in one meditation session. Each of these practices results in different subjective ASCs (as recently demonstrated by Woods et al. 2022) and subsequently involves different neural activity patterns (as reported by Lehmann et al. 2001, Amihai and Kozhevnikov 2014). In this review, we will use the gross categorization of FA, OM, LK, and ST practices, albeit its over-simplicity, in order to attempt categorical conclusions.

### Predictive processing accounts of meditation and their implications for complexity

The use of complexity-related measures in analyzing neural activity allows a link to currently leading predictive processing accounts of brain function. In this section, we outline proposed mechanisms of meditation which build upon the predictive processing framework and offer tentative hypotheses for the directionality of change in complexity in meditation in light of these hypotheses.

Predictive processing (Clark 2013, Hohwy and Seth 2020) posits that the brain constantly generates and updates a hierarchical generative model of the body and environment. Higher levels in the predictive hierarchy project predictions of the incoming input to lower levels, the lowest level being the senses. The brain strives to minimize prediction errors, which are the discrepancies between predictions and actual input, either by model updating or by action that changes the input to fit predictions (Parr et al. 2022). Prediction errors travel up the hierarchy and are given a precision-weighting based on the inverse variance of the signal (Friston 2010). Importantly, the top-down projection of expected precision is theorized to correspond to the process of attention allocation (Feldman and Friston 2010).

From a predictive processing perspective, meditation may involve the refinement of predictive models by bringing attention to the present moment and attenuating the influence of pre-existing biases and expectations. Through sustained practice, meditators may become more skilled at distinguishing predictions from sensory input, enhancing present-moment perception and reducing cognitive biases, and automatic thought patterns (Lutz et al. 2019). Furthermore, maintaining attention to sensory input in the present moment may increase precision weighting for bottom-up information, while the meditative stable posture may inhibit correction of prediction errors via motor action, requiring updates to priors instead (Pagnoni 2019). Another account (Laukkonen and Slagter 2021) proposes that as the depth of meditation increases, conceptual processing in the form of high-level priors gradually falls away, subsequently revealing a state of pure awareness, a process referred to as a “flattening” of the counterfactually and temporally “thick”^3^ self-model.

The theoretical frameworks described above do not explicitly generate hypotheses regarding the complexity of neural dynamics during meditation, and could be interpreted in either direction. On the one hand, meditation may lead to a relaxation of high-level priors and less suppression of prediction error through action, enabling greater bottom-up information flow, resulting in more intricate neural activity and higher complexity. This interpretation is similar to the “relaxed beliefs under psychedelics” (REBUS) theory (Carhart-Harris and Friston 2019), which is supported by ample empirical findings (e.g. Schartner et al. 2017, Timmermann et al. 2019, Mediano et al. 2024, Varley et al. 2020). On the other hand, the same process of “flattening” the counterfactual depth of the generative self-model, alongside attenuation of sensory information that supports the self-model (Limanowski and Friston 2020) may result in decreased complexity. This interpretation may also be supported by findings that meditation involves “switching off” neural networks supporting the narrative-self (Farb et al. 2007, Brewer et al. 2011, Garrison et al. 2015, 2015, Berkovich-Ohana et al. 2016), or attenuating networks supporting the embodied self (Dor-Ziderman et al. 2013, 2016). In light of these theories and the ambiguous hypotheses regarding changes in complexity in the meditative state offered here, we proceed to review the experimental literature of complexity measures in meditation.

## A scoping review of complexity in meditation

To investigate the current empirical evidence regarding complexity of neural activity in the meditative state (short-term) and trait (long-term) changes in meditators, we conducted a scoping literature review. Deolindo and colleagues (2020) provided a brief overview on studies of meditation through non-linear measures applied to EEG data, reviewing mixed empirical findings that suggest either an increase or decrease of complexity associated with meditation. Their conclusion was that the considered studies are not directly comparable because of the heterogeneity in designs and measures. Here we substantially broaden the amount of studies reviewed and aspire to provide a comprehensive framework for comparing these studies, that takes into account and explains the methodological differences as best as possible.

### Review protocol

Our review included searching the electronic databases PubMed and Google Scholar using the following query: (“meditation” OR “mindfulness”) AND (“complexity” OR “entropy” OR “fractal”). The inclusion criteria were sufficient sample size (*n* > 6), report of statistical analyses, description of the directionality of changes, and a relevant contrast (contrasting a similar condition between two groups or contrasting two conditions within one group).

We found 20 articles published in peer-reviewed journals, 10 conference proceedings and 1 PhD thesis. Out of these, we include 17 studies in this review, for which we report here only the significant results relevant to our review of complexity in meditation. We excluded three studies due to small sample size (*n* = 3: Davis et al. 2020; *n* = 2: Lin and Li 2017; *n* = 2: Pradhan and Narayana Dutt 1995), four studies that utilized complexity-related measures in meditation for machine learning classifiers and reported classification accuracy but did not report any descriptive analysis on the directionality of change in complexity (Korde et al. 2018, Han et al. 2020, Jachs 2022, Pandey et al. 2023), five studies which reported results but did not apply basic statistical significance testing (Harne 2014, Kamthekar and Iyer 2021, Kaur et al. 2017, Lo and Huang 2007, Pandey and Miyapuram 2021), and two studies contrasting a condition of experienced meditators during meditation vs. resting state of controls, which is an irrelevant contrast for our purposes^4^ (Huang and Lo 2009, Shaw and Routray 2016). Furthermore, we excluded one study comparing meditation to video watching (Davis et al. 2023). This may be a poor choice of contrast, as it has been shown that video watching induces a very large increase in LZc compared to resting state (Mediano et al. 2021), and substantially diminishes the measurable changes in LZc under LSD ingestion (Mediano et al. 2024). Given LSD’s robust impact on neural complexity, we suggest video watching likely obscures meditation’s subtler effects and is therefore a contrast unfit for our purposes.

All reviewed studies performed measurement of neural activity via M/EEG. A detailed review of all studies can be found in “Literature review” section, and a summary of the results and design of the reviewed meditation studies can be found in Tables 1, 2, and 3.

**Table 1.** Studies of meditation short-term effects in a within-subjects design.

No.	Reference	*N*	Experience	Meditation style	Category	Contrast	Measure	Change	Regions/ Frequency bands	Reported effect size	Cohen’s *d* (calculated)
1	Aftanas and Golocheikine (2002)	20	3–7 years	Sahaja Yoga	OM	Rest	DCx	Decrease	Midline Frontal & Central	*F* = −8.6	−0.927
2	Kakumanu et al. (2018)	21	Teachers: Av. 16.3 years, 15 349 hours	Annapana-Sati	FA	Rest	PE	Increase	Overall	*Z* = 1.28–1.38	0.284
				Vipassana	OM					*Z* = 1.28–1.38	0.292
				Metta-Bhavna	LK					*Z* = 1.28–1.38	0.284
				Vipassana	OM		HFD	Increase	Overall	Z = 1.18–1.29	0.271
		24	Beginners: Av. 2.2 years, 1 hours	Vipassana	OM	Rest	PE	Increase	Overall	*Z* = 1.28–1.38	0.265
							HFD	Increase	Overall	*Z* = 1.18–1.29	0.241
3	Vyšata et al. (2014)	22	>1000 h	Theravada	FA	Rest	Sevcik’s FD	Increase	Overall	N/A	**0.322**
							PE	Decrease	Overall		**−0.175**
					OM	Rest	PE	Decrease	Overall	N/A	**−0.399**
4	Kumar et al. (2021)	14	Av. 24 years	Rajayoga peaceful soul meditation	ST	Rest	MVMFE	Increase	Gamma band in frontal regions	*Z* = 1.6	0.428
				Rajayoga angelic consciousness meditation						*Z* = 2.668	0.713
5	Lu and Rodriguez-Larios (2022)	25	None	Breath attention	FA	Mind wandering	LZc	Increase	Left frontal; parietal & right frontocentral	N/A	**0.3865**
							SE				**0.4329**
							HFD		Overall	N/A	**0.543**
6	Walter and Hinterberger (2022)	30	Av. 20 years, 6498 h	Thoughtless emptiness	ST	Rest	DFA	Decrease	Overall	*d* =−0.37	−0.37
							HFD	Increase		*d* = 0.23	0.23
				Presence Monitoring	OM	Rest	DFA	Decrease	Overall	*d* = −0.49	−0.49
				Attention between the eyebrows	FA	Rest	MSE	Increase	Overall	sf = 1: *d* = 0.61 ; sf = 3: *d* = 0.48	0.545
							DFA	Decrease		*d* = −0.29	−0.29
7	Sik et al. 2017	11	None	Mindfulness based stress reduction (MBSR)	OM	Rest	WE	Decrease	Mid-frontal, mid-parietal & occipital	N/A	**−0.57**
8	Do et al. 2023	22	None	Mindfulness based stress reduction (MBSR)	OM	Podcast Listening	HFD	Increase	Overall	N/A	0.533
						Meditation week 4 vs. week 6					0.711
9	Young et al. (2021)	22	Av. 22 years, 21 934 h	Shamatha (FA), Vipassana (OM), Zazen(OM), Tonglen (LK), Dzogchen (ST), Deity Visualization (ST)	Mixed	Mind Wandering	LZc	Decrease	Overall	*F* = −5.24	−0.69
10	Irrmischer et al. (2018)	8	Av. 18 years	Breath attention	FA	Rest	DFA	Decrease	Theta, alpha, and beta bands	*t* = −3.4; *t* = −3.7; *t* = −4.2	−1.88
		10	None	Breath attention	FA	Rest	DFA	Increase	Alpha, beta, gamma bands	*t* = 2.6; *t* = 2.9; *t* = 2.7	1.22
		20	Av. 10.8 years	Breath attention	FA	Rest	DFA	Decrease	Delta, theta, alpha, beta and gamma bands	*t* =−2.4; *t* = −2.7; *t* = −2.7; *t* = −2.8; *t* = −2.7	−0.84
						Pre-post 1 year intervention		Decrease	Alpha band	*t* = −2.5	−0.79
11	D’Andrea et al. (2024)	10	Av. 15.5 years, 14 765 h	Theravada	FA	Rest	DFA	Decrease	Overall, derived from MEG microstate sequences	*t* = −4.15	−1.86
							LZc	Increase		t = 5.4	2.42
					OM	Rest	DFA	Decrease		*t* = −3.41	−1.53
							LZc	Increase		*t* = 14.46	6.47
12	Anasi et al. (2018)	19	None	Mindfulness based stress reduction (MBSR)	OM	Pre-post 2 months intervention	HFD	No Significant difference	Overall	N/A	N/A

Where information for two adjacent cells is the same, cells are merged. Calculated Cohen’s d: Where in bold, no effect sizes or standard deviation (SD) of difference were reported, so Cohen’s d was calculated based on groups mean and SD assuming independent samples. Where effect was shown within the same group and condition for different frequency bands/regions, the calculated Cohen’s d is an average of the effect sizes. For Kakumanu and colleagues (2018), effect sizes were reported only through scalp topography graphs, hence a range is written, and the calculated Cohen’s d is a weighted average of the effect sizes seen on the scalp topography.

**Table 2.** Studies of meditation state (short-term) effects in a between-subjects design.

No.	Reference	N	Experience	Meditation Style	Category	Contrast	Measure	Change	Regions/ Frequency Bands	Reported Effect Size
3	Kakumanu et al. (2018)	24 Beginners vs. 22 Seniors +21 Teachers	Teachers: 16.3 years, 15 349 hs, Seniors: 13 years, 10 364 h, Beginners: 2.2 years, 1000 h	Annapana-Sati Vipassana Metta-Bhavna Annapana-Sati Vipassana Metta-Bhavna	FA OM LK FA OM LK	Begginers vs. Teachers & Seniors	HFD PE	Decrease	Overall	N/A
11	Irrmischer et al. (2018)	8 Meditators, 11 Controls	Meditators: av. 18 years, Controls: none	Breath attention	FA	Meditators (FA-Rest) vs. Controls(FA-Rest)	DFA	Larger Decrease	Delta	*t* = 3.2
		20 Meditators, 10 Controls	Meditators: av. 10.8 years, Controls: none					Decrease for Meditators, increase for Controls	Delta; alpha; beta; gamma	*t* = 2.7; *t* = 3.1; *t* = 4.5; *t* = 3.3
16	Martínez Vivot et al. (2020)	16 Meditators, 16 Controls	Av. 15 475 h	Himalayan Yoga	FA				High gamma	*Z* = 2.12 *Z* = 2.28
			Av. 9201 h	Vipassana	OM	Controls FA	SE	Increase	Alpha & low gamma	*Z* = 2.65
			Av. 2625 hours	Isha Shoonya Yoga	OM			Increase, not significant	N/A	N/A
17	Singh et al. 2023	16 Meditators, 16 Controls	Av. 15 475 h	Himalayan Yoga	FA	Controls FA	SE	Increase	Gamma band, occipito-parietal regions	N/A
							HE SE	Decrease Increase	Alpha band, frontal & parietal regions;	
			> 1 year, 2 h a day	Hare Krishna	ST	Controls FA	HE	Decrease	gamma band, occipito-parietal regions	N/A

The “Number” column continues and corresponds to the count from Table 1. Where information for two adjacent cells is the same, cells are merged.

**Table 3.** Studies of meditation trait (long-term) effects.

No.	Reference	*N*	Experience	Category	Trait Condition	Contrast	Measure	Change	Regions/ Frequency Bands	Reported Effect Size
3	Kakumanu et al. (2018)	24 Beginners vs. 22 Seniors +21 Teachers	Teachers: 16.3 years, 15 349 h, Seniors: 13 years, 10 364 h, Beginners: 2.2 years, 1000 h	FA + OM + LK	Rest	Begginers vs. Teachers & Seniors	PE HFD	Decrease	Overall	N/A
11	Irrmischer et al. (2018)	20	Av. 10.8 years before; Av. 11.8 years after	FA	Rest	Pre-post 1 year intervention	DFA	Increase	Delta; beta; gamma	*T* = 2.6; *t* = 2.4; *t* = 2.5
13	Gupta et al. (2021)	20	None	FA	Cognitive task	Pre-post 2 month intervention	HFD	Decrease	Overall	N/A
15	Tibdewal et al. (2022)	25	None	N/A	Rest	Pre-post 1 month intervention	SE	Increase	Delta & beta bands: frontal lobe	N/A
								Decrease	Theta & alpha bands: frontal lobe	

The “Number” column continues and corresponds to the count from Tables 1 and 2. Where information for two adjacent cells is the same, cells are merged.

### Analysis protocol

The first observation in our review is that there is substantial discrepancy between the reviewed studies in the designs, analysis methods, measures used, etc. Therefore, a comparison of the results of these studies is not straightforward, and a rigorous mathematical meta-analysis is not possible. Hence, we conduct our analysis according to the following protocol. For the results of this analysis see “Analysis and results” and the Supplementary information.

First, we split the reviewed studies based on study design, into meditation state (short-term) and trait (long-term) studies.^5^ Second, for each design category we offer an analysis based on categorization of complexity-related measures, categorization of meditation style, and meditation experience. For the first design category (within-subjects meditative state studies) we conduct an effect size analysis comparing average effect sizes between families of complexity measures, meditation experience, and also different study design/preprocessing factors including number of M/EEG channels, sampling rate, filtering parameters, and number of data points per complexity estimate.

Categorization of complexity measures follows our identification of two facets of complexity previously described, dividing the measures into two families: entropy and fractal dimension measures. A description of each of the reviewed measures can be found in the Appendix.

The “entropy measures” family includes Shannon entropy, sample entropy (SE), multiscale entropy (MSE), permutation entropy (PE), wavelet entropy (WE) and minimum variance modified fuzzy entropy (MVMFE). These measures all relate to the Shannon entropy of the time series, yet estimate informational entropy via different manipulations and methods (see the Appendix for an overview). Closely related to the above is Lempel–Ziv complexity (LZc) which counts the number of distinct patterns in a binarized time series, converging to the entropy rate of the process generating the signal.

The “fractal dimension measures” family includes Higuchi’s fractal dimension (HFD) and Sevcik’s fractal dimension (SFD) which directly treat the time series as a fractal pattern, and dimensional complexity (DCx) which estimates the amount of squares/cubes the trajectory of the signal in phase space fills as it evolves. We also include measures of LRTC which came up in our search: Hurst’s exponent (HE) and detrended fluctuation analysis (DFA). Hurst’s exponent quantifies the rate at which autocorrelations between value pairs in the time series decay as the time distance between the pair increases, and DFA computes a scaling exponent which is an estimation of the Hurst exponent by dividing the time-series into segments and calculating the amount of fluctuation in the data as a function of segment size. As mentioned previously, under the assumption of a self-similar time series,^6^ the Hurst exponent is directly related to the fractal dimension by FD = 2 HE for 1 < FD < 2 (Mandelbrot 1985). Therefore, it should be kept in mind when reading the review that under the assumption of self-similarity, when a decrease in DFA or HE is demonstrated this actually entails an increase in the fractal dimension, and vice versa. Specifically in the case of EEG, a signal that contains both fractal and oscillatory aspects, it has been shown that HFD over-estimates the fractal dimension, while DFA under-estimates it (Krakovská and Krakovská 2021).

Categorization of meditation style follows our proposed typology in “Meditation — a gross typology” section. We divide the various meditation techniques in the reviewed studies into focused attention (FA), open monitoring (OM), loving-kindness (LK) and self-transcendence (ST). We analyze meditation experience by years of meditation, and rely either on a direct report in the reviewed study (where available), or estimate through reported meditation hours (assuming 200 h per year, where average practice per day was not reported).

For effect size estimation, Cohen’s *d* was calculated with an online tool (https://www.campbellcollaboration.org/calculator/, based on the book “Practical Meta-Analysis” by Lipsey and Wilson 2001) from effect sizes (*t*, *F*, *z*, or *d*) where reported, and from means and standard deviations (SD) where effect size was not reported in the reviewed studies. In the latter case, as the SD of the difference between conditions was not reported, Cohen’s *d* was calculated assuming independent samples (unpaired *t*-test). This assumption does not affect the directionality of the results, but typically produces a smaller effect size than when assuming dependent samples.

**Figure 2:**
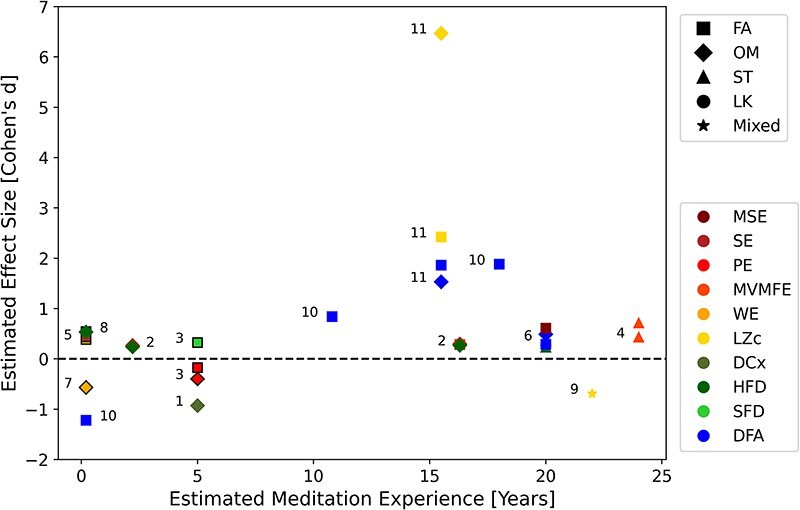
A summary of the within-subject studies comparing meditative state to a control condition of rest or mind-wandering. Each data-point represents a unique combination of group, meditation style, and measure; therefore, some studies contribute more than one data point. Colors indicate measures, and shapes indicate meditation styles. Data points are annotated with the number of their corresponding study in Table 1. Data-points with no outline indicate an effect size based on dependent samples (paired *t*-test), data points with black outline indicate estimated effect size assuming independent samples (unpaired *t*-test). It should also be noted that as the Hurst exponent (HE) relates to fractal dimension (FD) through the formula FD=2-HE for 1

This calculation of Cohen’s d was done for each unique combination of complexity measure and meditation style and group in the reviewed meditation state studies (Table 1), to obtain the data presented in Fig. 2. To compare average effect sizes between complexity measure families, we then averaged effect sizes taking one data point for each measure and group, averaging across meditation styles to create each data point. In principle, there are several ways to create the data points for averaging and we chose the aforementioned scheme to account for different measures therefore, this analysis should be read as a general way of showing numerical trends and not as a rigorous mathematical meta-analysis. To compare average effect sizes between different study design and preprocessing factors, we averaged the estimated effect size within each study (across measure type and meditation style) to obtain one data point for each study. In this case, we chose this scheme in order to give an equal weight to each study in the overall averaging.

### Literature review

#### Studies of the meditative state (short-term effects)—within-subject design

First, we review studies contrasting meditation with a control condition, typically eyes-closed rest or mind-wandering, in a within-subject design. As the variability in baseline neural complexity is large (see preprint, Mediano et al. 2021), examining changes in complexity-related measures in a within-subject design is preferable. For a summary of results in this category, please refer to Table 1.

Six studies demonstrated an increase in measures for the meditative state compared to control conditions. For meditation-naive subjects performing FA meditation, Lu and Rodriguez-Larios (2022) report an increase in LZc, SE and HFD compared to mind-wandering. Four studies demonstrated an increase in measures in experienced meditators for various meditation styles compared to resting state. The first study demonstrated an increase in MVMFE in highly experienced Rajayoga (ST) meditators (Kumar et al. 2021), the second demonstrated an increase in HFD for a ST meditation and an increase in MSE for FA meditation performed by highly experienced meditators (Walter and Hinterberger ), the third demonstrated an increase in PE and HFD for FA, OM, and LK meditation conditions for highly experienced meditators, and an increase in HFD and PE for the OM meditation in beginner meditators (Kakumanu et al. 2018), and the fourth study reported an increase in LZc for both FA and OM meditation performed by highly experienced Theravada meditators (D’Andrea et al. 2024). Additionally, one study demonstrated an increase in HFD in meditation compared to podcast listening for meditation-naive subjects at week 6 of a mindfulness-based stress reduction (MBSR) course, and an increase in HFD in the meditation condition from Week 4 to Week 6 of the course (Do et al. 2023). In addition, three studies demonstrated a decrease in the Hurst exponent, as estimated using DFA in meditation compared to resting state in experienced meditators. The first study reported a decrease in FA meditation for two independent groups (Irrmischer et al. 2018), with one of the groups showing an enhanced effect after an additional year of meditation training, the second study reported decreases for different meditation conditions including FA, OM, and ST (Walter and Hinterberger ), and the third study reported a decrease for both FA and OM meditation (D’Andrea et al. 2024).

Three studies demonstrated a decrease in different measures for the meditative state compared to control conditions. One study demonstrated a decrease in LZc in highly experienced meditators performing different styles of meditation compared to mind wandering (Young et al. 2021). Two studies demonstrated a decrease in different measures for the meditative state compared to resting state. The first demonstrated a decrease in DCx in experienced Sahaja Yoga (OM) meditators (Aftanas and Golocheikine 2002), and the second demonstrated a decrease in WE in novice meditators undergoing an MBSR course (Sik et al. 2017). Additionally, Irmischer and colleagues (2018) report an increase in DFA in a FA meditation performed by meditation-naives, compared to resting state.

Two studies reported inconsistent results within a single dataset. The first study demonstrated an increase in SFD and a decrease in PE for an FA meditation and a decrease in PE for an OM meditation performed by experienced meditators (Vyšata et al. 2014). The second study reported a change in HFD for meditation-naives performing an OM meditation before and after an MBSR course, showing an increase for some EEG channels and a decrease for others, with no significant overall difference (Anasi et al. 2018).

#### Studies of meditation state (short-term effects)—between-subjects design

Next, we review studies with between-subject designs, comparing the meditative state between meditators and controls. For a summary of results in this category please refer to Table 2.

We found two studies demonstrating an increase in measures when comparing a meditation condition of experienced meditators with novices (i.e. inexperienced controls). Martínez Vivot and colleagues (2020) demonstrated higher SE in meditation of Himalayan Yoga (FA) and Vipassana meditators (OM) compared to an FA meditation performed by controls. The second study utilized the same dataset for the Himalayan Yoga and control groups, confirming the result of higher SE in the meditators than in controls, and reporting lower HE for the same comparison. This study also reported higher SE and lower HE for an additional Hare Krishna mantra (ST) group, compared to controls FA (Singh et al. 2023).

On the other hand, Kakumanu and colleagues (2018) demonstrated that for all meditation conditions (OM, FA, LK), overall HFD and PE values were lower for seniors and teachers when compared to beginner meditators.

Finally, Irmischer and colleagues (2018) also contrasted the difference between meditation and rest, between the meditator and control groups. They demonstrate that for meditators there is a stronger decrease in DFA during FA meditation than for the controls doing the same meditation.

#### Studies of meditation trait (long-term effects)

Studies in which analysis were carried out during resting state or cognitive tasks, comparing experienced meditators to novice meditators or comparing the same participants before and after a meditation intervention, are regarded as trait studies. For a summary of results in this category, please refer Table 3.

Kakumanu and colleagues (2018) compared resting state between experienced meditators (seniors and teachers) and beginner meditators matched for age, gender and other demographic factors, and reported that HFD and PE values were considerably lower for the experienced group. Tibdewal and colleagues (2022) compared resting-state of meditation-naives before and after a one-month meditation intervention, and reported a post-intervention increase in SE for the delta and beta bands and a decrease for the theta and alpha bands. Irmischer and colleagues (2018) examined resting state of experienced meditators before and after a one-year FA meditation intervention. They demonstrated a post-intervention increase in resting-state DFA. Finally, Gupta and colleagues (2021) compared a cognitive task performed by meditation-naives before and after a 2-month FA meditation intervention, and reported a post-intervention decrease in HFD.

### Analysis and results

#### Studies of the meditative state (short-term effects)—within-subject design

For the main design category of within-subject meditative state studies, we hereby present an analysis of the reviewed studies based on meditation experience and style, family of complexity measures and preprocessing pipelines. Although the reviewed studies show variation in the directionality of changes in complexity, we have identified a robust trend towards increased complexity during the meditative state when compared to waking rest or mind-wandering, with the majority of studies painting to this direction (see Table 1). This effect of increased complexity in the meditative state is more pronounced for experienced meditators, and is present regardless of type of complexity measure used, meditation style, or preprocessing pipeline, as will be subsequently elaborated. For a detailed visualization of the estimated effect sizes in the meditation vs. baseline comparison, as a function of meditation experience, see Fig. 2.

We can see that meditation experience is an important factor when interpreting results of our reviewed studies. When meditation experience exceeds 5 years, almost all studies show an increase, while for less experienced meditators results vary. On the other hand, meditation style is not very informative when attempting to discern factors of variation in the direction of change. ST meditation consistently produces an increase in the measures, LK which was investigated in only one study shows an increase, yet OM and FA meditation show both.

The family of entropy measures (yellow–red colors) show an increase in the majority of studies, with an average effect size of *d* = 0.717. When accounting for meditation experience ≤5 years these measures produce an average effect size of *d* = −0.001 and >5 years an average effect size of *d* = 1.201. Fractal dimension measures (green colors) show an increase in the majority of studies, with an average effect size of *d* = 0.173, with *d* = 0.142 for inexperienced meditators (≤5 years) and *d* = 0.25 for experienced meditators (>5 years). DFA shows decrease in the majority of studies (corresponding to an increase in fractal dimension), with an average effect size of *d* = −0.755, while the average effect size is *d* = −1.037 for experienced meditators and an increase (corresponding to a decrease in fractal dimension) for meditation-naives (shown in one study) with effect size of d = 1.22.

We furthermore attempted to analyze the results based on different data acquisition procedures and preprocessing pipelines applied in the studies. In principle, factors such as sampling frequency, number of EEG sensors, frequency filters, cleaning of the EEG signal, data points per epoch for measure calculation and other preprocessing such as a detrend of the data and Hilbert transformation of the signal prior to measure calculation can have a substantial impact on measure outcome (Durschmid et al., 2020, Lau et al. 2022). When analyzing the results in light of these factors, we could perform only a limited analysis due to the fact that not all studies fully reported their pre-processing pipelines, and high heterogeneity in some of the study design/pre-processing factors. Therefore, we analyzed the results only for number of channels in the M/EEG system, sampling rate, value of low-pass filter, and number of data points in each epoch used to calculate the various measures. A graphic representation of the effect sizes as a function of the value of these factors can be seen in Supplementary Figures 1–4 and data used for these figures can be found in Supplementary Table 1.

Our results show that that generally in studies where M/EEG systems with a high number of channels were used, complexity in meditation was higher than rest, while in systems with a small amount of channels results were ambiguous. Average effect size in studies using 32 channels or less was *d* = 0.05, while for studies using 62 channels or more average effect size was *d* = 0.67. A similar trend arose for sampling frequency, where higher sampling frequencies resulted in a more pronounced effect of increase in complexity during meditation. Average effect size in studies that sampled at 256 Hz or below was *d* = 0.19 while for 500 Hz and above average effect size was *d* = 0.79. The value of the lowpass filter applied also seemed to affect results: studies applying a lowpass filter of 45 Hz or less had an average effect size of *d* = 0.71, and studies applying a lowpass filter of 60 Hz or more on average showed a reduced complexity in the meditative state, with an average effect size of *d* = −0.236. Number of data points in each epoch used for measure calculation did not seem to affect the average effect sizes or directionality of results.

#### Studies of meditation state (short-term effects)—between-subjects design

Here the small number of studies (*N* = 4, see Table 2) does not allow for a thorough analysis of the results (as in “Studies of the meditative state (short-term effects)—within-subject design”section ) as well as impede reaching a definite conclusion. Furthermore, we deem this type of contrast, comparing the meditative state between subjects less relevant for our purposes, as it does not shed light on the differences in complexity between meditation and baseline. Nevertheless, here we identify a trend towards higher complexity during the meditative state in experienced meditators compared to controls, with three studies out of four pointing to this direction (see Table 2).

#### Studies of meditation trait (long-term effects)

Here the small number of studies (*N* = 4, see Table 3) does not allow for a thorough analysis of the results (as in “Studies of the meditative state (short-term effects)—within-subject design” section) or reaching a definite conclusion. Nevertheless, the results here seem less ambiguous, showing a decrease in trait entropy and fractal dimension measures, as a function of meditation proficiency in three out of four studies, and mixed results in one study (see Table 3). Therefore, we can conclude with some certainty that this trend of reduced baseline complexity in experienced meditators is a real effect that requires further experimental validation. A potential limitation of this finding is the influence of age-related changes in complexity, which increase from youth to middle age and decline in old age (Zappasodi et al. 2015, Ishii et al. 2017). However, this limitation seems unlikely here, as the studies comparing resting-state complexity (Table 3) either compared age-matched groups differing in meditation proficiency (Kakumanu et al. 2018), or compared individuals before and after mediation training of 1 month (Tibdewal et al. 2022), 2 months (Gupta et al. 2021), and 1 year (Irrmischer et al. 2018)—durations unlikely to significantly affect complexity due to aging. Therefore, although the small number of studies precludes a definite conclusion, we believe the observed reduction in baseline complexity is indeed linked to meditation proficiency rather than aging.

## Discussion

### Overview of results

In this paper, we provided an organizing framework for the study of complexity of neural activity in meditation and reviewed the empirical work that has accumulated in this field. As studies varied in their design, applied measures, and preprocessing pipeline, ambiguous findings were reported in the reviewed studies. Albeit the seemingly ambiguous findings, through the organizing framework that we have proposed, and through a pooling of the results of the reviewed studies, our review uncovers a convergence toward identifying higher complexity during the meditative state when compared to waking rest or mind-wandering, and decreased baseline complexity as a trait following regular meditation practice.

In order to organize the results, we divided our review by differentiating between state from trait effects, different families of complexity-related measures, meditation styles and experience, and also preprocessing steps. Our findings show that the most meaningful differentiation regarding changes in complexity during or following meditations were meditation state-vs.-trait, and meditation experience. The meditative state showed a trend of increased complexity compared to waking rest or mind-wandering, while trait baseline complexity decreased with greater meditation proficiency. Notably, the effect of increased complexity in meditation vs. rest was consistent across studies in experienced meditators, whereas inexperienced meditators showed variable results, as elaborated below. Furthermore, we found that several study design and preprocessing factors such as filter value, number of M/EEG channels and sampling frequency tend to affect results of complexity measures, yet these effects are not readily interpretable due to high heterogeneity and require more studies to reach a definite conclusion. In our analysis, meditation style seemed not to be a meaningful factor of variation, although this lack of effect may be due to an insufficient number of studies for comparison, specifically since the categories of LK and ST together were present in only four studies.

Our analyses support a trend towards higher complexity in the meditative state compared to resting-state wakefulness and mind-wandering, with a majority of the meditation-state studies reviewed pointing to this direction. This increase is more prominent in studies examining experienced (>5 years) meditators, and more consistent for the fractal dimension measures than for entropy measures.

Our analyses also showed that during meditation proficient meditators, compared to novices, tend to have higher neural complexity—albeit one study (Kakumanu et al. 2018) showing opposite results. In contrast, studies of trait (long-term) effect consistently show a reduction in baseline complexity as a function of meditation proficiency, when comparing individuals before and after meditation training in a within-subject design, and when comparing between individuals with low- and high-meditation proficiency.

This finding of elevated complexity during the meditative state and decreased complexity as a trait in experienced meditators has also received direct empirical support in a recent fMRI study preprint (Atasoy et al. 2023).

Overall, the results of our review suggest that meditation practice may operate in a way that increases complexity of neural activity during the practice, and decreases baseline complexity as a function of meditation proficiency. In the following, we first discuss these tentative findings in light of predictive processing theories of meditation and in relation to the psychedelic state, and finally offer guidelines for future research in the field.

### Results in light of predictive processing accounts of meditation

The trend towards increased neural complexity in the meditative state (compared to mind-wandering and rest) may suggest that increases bottom-up information flow. This interpretation stands in stark contrast with the idea that a “switching off” of neural networks during meditation could be thought of as leading to lower complexity in the neural dynamics. That said, one possible way to integrate the idea of a “switching off” of neural networks with the observed trend of higher complexity could be to argue that the prediction-based control of high-level networks may regularly suppress information conveyed by lower-level systems (e.g. systems conveying sensory information), but this suppression may be weakened during meditative states. This argument is in line with findings showing decreases in DMN activity during meditation (Farb et al. 2007, Brewer et al. 2011, Garrison et al. 2015).

The result of our analysis is also consistent with claims that, in meditation, prediction errors travel farther up the predictive processing hierarchy thanks to a combination of (i) a relaxation of high-level priors, and (ii) the refraining from physical action or mental gestures that would otherwise minimize prediction errors (Lutz et al. 2019). Under the assumption that information about the world is conveyed in the form of prediction errors, it can be argued that more information is processed in the brain during meditation—and indeed, this has been phenomenologically demonstrated by Petitmenging and colleagues (2018). Moreover, the process of relaxation of high-level priors and a refraining from action to minimize prediction error can be seen as somewhat orthogonal to the basic tendency to minimize prediction errors. This may allow for a temporary state in which the brain is forced to account for information that is usually repressed. Interestingly, this interpretation directly relates to some of the traditional definitions of the purposes of meditation (Harvey 2015), as in Buddhism, meditation is used for “a direct experiential realization of the nature of reality” (Dreyfus and Thompson 2007), which might correspond to the relaxation of priors. In a similar vein, equanimity, defined as an even-mindedness toward all experiences regardless of their origin or affective valence (Desbordes et al. 2015), may correspond to the process (and long-term realization) in which prediction errors are less automatically suppressed.

It is worth noting that although here we have chosen to focus on predictive processing as a framework for interpreting the empirical results, future work could investigate how meditation relates to other theories in the field of consciousness research such as Integrated Information Theory (Tononi et al. 2016, Albantakis et al. 2023) and Global Neuronal Workspace (Dehaene et al. 1998, Mashour et al. 2020).

### Comparison to the psychedelic state

As previously noted, the psychedelic state involves ASCs which have been robustly linked to an increase in complexity compared to resting state. Due to this fact, and to several phenomenological similarities between meditation and psychedelics (Millière et al. 2018, Letheby 2022), we deem the psychedelic state a relevant ASC to be compared to the meditative state in relation to changes in complexity. Our analysis suggests that the meditative state, similar to psychedelic ingestion, is characterized by higher complexity compared to normal wakefulness. In the entropic brain hypotheses, Carhart-Harris and colleagues (2014) suggest that in normal waking consciousness, the brain is operating in a subcritical regime, and that states of primary consciousness (such as early psychosis, sensory deprivation, dreaming, psychedelics, and infant consciousness) may push neural dynamics closer to the point of criticality. Following this line of reasoning, it could be argued that meditative states also involve states of primary consciousness, corresponding to a “beginner” state of mind in which previously formed biases are attenuated, allowing for a more present-focused state (Austin, 1999, Suzuki, 2020).

The entropic brain hypothesis also makes a prediction that an increase in entropy surpassing the critical point may lead to a loss of consciousness. However, to the best of our knowledge, no ASCs have been yet observed reflecting this change of trend. Building on our finding that the meditative state tends to entail increased entropy, and the fact that that meditation is a gradual process that involves a continuous practice, we propose a candidate ASC for the category of loss of consciousness due to an increase in entropy. The ASC we propose is the state of Nirodha-Samapatti, which is a pinnacle of meditation practice and is phenomenologically characterized by an increasing sense of meditative depth, followed by a transient cessation in conscious experience (Sharp 2011, Berkovich-Ohana 2015, Laukkonen et al. 2023). The study of this ASC, and the process leading to it, in the context of complexity of neural activity, is a promising avenue for future work, which may propose a first example of passing beyond the hypothesized critical point proposed by the entropic brain hypotheses.

Another interesting phenomena arising from our analysis is the reduction of baseline complexity as a function of meditation experience. Carhart-Harris and Nutt (2017) and Carhart-Harris and Friston (2019) suggest that the transient increase in entropy induced by psychedelics enables the neural system to explore new regimes of information processing, potentially leading to more efficient functional pathways that may persist after the experience (Hipólito et al., 2023). We see the results of increased complexity during the meditative state, and decreased baseline complexity as a function of meditation experience as a support to this notion, with numerous potential mental-health implications.

### Limitations and guidance for future studies in the field

Firstly, complexity-related measures are in principle sensitive to preprocessing steps, and especially to the time-window used for calculation, the signal to noise ratio (Lau et al. 2022), and the values of frequency filters (Dürschmid et al. 2020). In our analysis, we found that indeed several study design and preprocessing factors tend to affect the directionality of changes in complexity in the reviewed studies. Therefore, we call for more open data practices, which will allow a re-examination of reported results under different pipelines. In the case that sharing data is not possible, we encourage researchers to fully report the study design, especially the meditation instructions or phenomenology if it exists, and the employed data preprocessing pipelines. While some studies did report these in detail, this practice is still not widely adopted, and the lack of it adds unnecessary difficulties when attempting to reach overarching conclusions.

Second, as complexity measures are sensitive to global states of consciousness, we encourage researchers to carefully choose the contrast to the meditative state of interest. As previously discussed, unlike eyes-closed rest, immersive states such as video watching (Davis et al. 2023) may obscure the effects of meditation on neural complexity and therefore if such states are of interest, they should be complemented with additional baseline contrasts (e.g as in Walter and Hinterberger ).

Thirdly, as previously elaborated, the notion of complexity is (to date) more a set of overarching principles rather than a single well-specified measurable property. Consequently, we encourage researchers to take caution when interpreting results of complexity-related measures, bearing in mind that each measure may only be able to capture aspects of a specific facet of a complex system and that the translation of their conceptual differences to measurable differences remains an interesting avenue for future research. Notably, although fractal dimension and entropy measures are conceptually distinct, in practice their application to time-series data in many cases yields similar behaviour (see Fig. 1). Among the reviewed studies that have utilized both types of measures, in three studies (Walter and Hinterberger, Kakumanu et al. 2018, Lu and Rodriguez-Larios 2022, ) these measures exhibited highly similar behavior, while only in one study did a fractal dimension and entropy measure show opposite direction of change in meditation vs. rest (Vyšata et al. 2014). In light of these issues, we encourage researchers to apply a predefined set of measures when attempting to estimate complexity, as well as analyze the dependencies and correlations that may exist between these measures, as discussed in Walter and Hinterberger (2022). In particular, the following is a set of measures that we deem as useful: MSE, LZc, and HFD, as each of these measures theoretically captures a complementary aspect of complexity.

Additionally, it is worth keeping in mind that changes observed in complexity-related measures may correlate with spectral power. Some of the reviewed studies (Walter and Hinterberger; Kakumanu et al. 2018, ) investigated this relation and found various correlations between complexity measures and spectral power in the meditative state. Generally, complexity measures were positively correlated with beta (coefficients ranging from *r* = 0.13 to *r* = 0.52) and gamma band power (*r* = 0.37 to *r* = 0.72), while the alpha band showed negative correlations (*r* = −0.11 to *r* = −0.58), and the delta and theta bands showed both directions of correlation. A theoretical discussion of the relationship between complexity measures and spectral power is out of the scope of this paper, yet considering their apparent inter-relation we wish to point this as a potent direction for future theoretical and empirical research. Efforts as such could benefit from computing both spectral and complexity-related measures and contrasting the variance explained by each of these [as done systematically by Donoghue and colleagues (2024)], or by applying estimators that inherently disentangle spectral power from complexity effects, such as the one presented by Mediano and colleagues (2023).

Finally, analyzing the results according to our categorization of meditation styles failed to account for differences in results. Although this lack of effect may result from the limited number of studies as previously discussed, if this is not the case, this finding may have at least two interpretations. Either complexity-related measures are able to characterize meditation beyond the differences in meditation techniques, or this categorization is gross and does not truly reflect the actual experience of meditators (especially for novices). Confirming or rejecting the first interpretation is an interesting avenue for future experimental work that may directly compare different meditation styles. The second interpretation points toward the deeper challenge of bridging first-person experience (and reports of the experience) and third-person measurements. This challenge has been discussed thoroughly in the literature, and a research program that we regard as particularly promising is neurophenomenology (Varela 1996).

Thus, considering the important variance introduced by the wide range of meditation techniques, and given that our analysis shows that a categorization of meditation styles fails to account for discrepancies between studies, we recommend neurophenomenology as a framework to aid bridging the gap between subjective and objective data. Phenomenological studies can be developed through many methods. For example, deep phenomenology allows us to go beyond classical categorizations of meditation and address the specific experience of participants (e.g. Nave et al. 2021). Also, the use of neurophenomenology in the study of ASCs has been strongly advocated by Timmerman and colleagues (2023), and a practical guide for some strategies of application was offered by Berkovich-Ohana and colleagues (2020). Some examples of such methods include micro-phenomenology (Petitmengin 2006), temporal experience tracking (Jachs, 2022)), descriptive experience sampling (Hurlburt and Akhter 2006) and even repeatedly introducing a simple question during measurement of neural activity (Lu and Rodriguez-Larios 2022). Considering the range of choices, choosing a neuro-phenomenological method for investigation should take into account the following aspects: depth of reports, practical implications, relevance to the time-course of the experience and the risk of biased information.

We hope that this review will inform the growing field of applying complexity-related measures to the study of meditation and its underlying neural activity, and will serve as an organizing back-bone for future studies, as well as inspire the use of open data and neurophenomenology, to enable reaching wider conclusions.

## Supplementary Material

niaf013_Supp

## Data Availability

There are no new data associated with this article as this is a review article. All data are available in Tables 1–3. If codes are required, please add that codes will be shared upon appropriate request.

## Footnotes

1 To be mathematically precise, entropy rate—see Mediano and colleagues (2023).

2 This theorem shows that the entropy is the number of fair coins that are needed to simulate a variable with an equivalent amount of uncertainty, or, put simply, the number of yes/no questions that are needed in average to reveal the value of the variable in question (Cover and Thomas 2012).

3 A generative model aimed at predicting the causes of sensory inputs is said to be temporally “thin” and concrete on the lower (sensory) levels of the hierarchy and more abstract and temporally “thick” (making inferences about the past and predictions about the future and generalizing the present moment) in higher levels (Friston and Sporns 2008; Corcoran et al. 2020, Laukkonen and Slagter 2021).

4 A contrast that compares both different groups and different conditions does not allow to discern if the measured changes in complexity-related measures arise from the accumulating long-term effect of meditation, which requires a contrast of the same condition between groups, or from the short-term effect of meditation, which requires a contrast of different conditions within the same group.

5 Note that several studies examine both state and trait effects of meditation, and therefore appear in both categories.

6 It should be noted that for more general stochastic processes this relation is broken, and the Hurst exponent and fractal dimension can be independent of each other (Gneiting and Schlather 2004).

## Appendix

To facilitate an easier reading of the review, here we provide a brief description of the complexity measures considered in the studies reviewed. For a complete description of each measure, please refer to the relevant article in which the measure was formulated, or to the relevant study in the review utilizing the measure.

**Entropy and related measures**

Shannon Entropy (Shannon 1948) is a measure of the uncertainty associated with a random variable. It quantifies the average amount of information that is gained when an observed measure the variable in question, or equivalently, the information needed to describe or predict it. Shannon Entropy is calculated by taking the average value of the logarithm of the probability of each possible event.

Permutation Entropy (PE, Bandt and Pompe 2002) is a measure that quantifies the diversity of patterns of a time series by analyzing the distribution of ordinal sequences, which are determined by the relative orderings of data points within a sliding window. PE then calculates the Shannon Entropy of the ordinal pattern distribution, providing a measure of the complexity or unpredictability of patterns observed on the time series.

Sample Entropy (SE, Richman and Moorman 2000) is a measure similar to PE, also used to quantify the diversity of patterns in time series. It calculates the probability of finding repeating patterns of a certain length within the data by comparing the similarity of overlapping subsequences. The Shannon Entropy is then calculated based on these probabilities to determine the SE value.

Multiscale Entropy (MSE, Costa et al. 2002) is an extension of SE, which calculates SE at different multiple levels of down-sampling or smoothing of the time series, choosing different values for a scaling factor. MSE thus provides a scale-dependent assessment of irregularity, allowing the identification of complex dynamics at different temporal resolutions.

Wavelet Entropy (WE, Zheng-you et al. 2006) is a measure that combines the concepts of wavelet analysis, using wavelet decomposition to capture both frequency and temporal information of the data and calculating the Shannon entropy of the resulting wavelet coefficients. Wavelet Entropy can reveal the complexity of different frequency components and their interactions within the time series.

Minimum Variance Modified Fuzzy Entropy (MVMFzEn, Raghu et al. 2018) combines fuzzy sets and Shannon entropy to capture both fuzziness and irregularity in the data. Fuzziness represents uncertainty or vagueness, indicating the lack of clear boundaries when assigning membership to categories or sets. It is calculated by assessing the ambiguity or overlap between different membership values. Irregularity refers to randomness or the absence of a predictable pattern. MVMFzEn incorporates a modification to enhance robustness by reducing sensitivity to outliers and noise.

Lempel–Ziv Complexity (LZc, Lempel and Ziv 1976) measures the complexity or compressibility of a binary string by identifying repeated patterns. It quantifies the number of distinct patterns found in the data. For estimating the complexity of a non-binary time series, such as EEG neural activity, a threshold is defined for binarization, usually chosen as the mean of the detrended time series. Under the condition of stationarity of the data, the LZc can be used as an effective estimator of the entropy rate of the time series (Mediano et al. 2023).

**Fractal dimension and related measures**

Higuchi’s Fractal Dimension (HFD, Higuchi 1988) is a method used to estimate the fractal dimension of a time series. This approach analyzes the scaling behavior of the curve through a process of dividing the time series into shorter segments and measuring the average length of the curve in each segment. The fractal dimension is then obtained by fitting a linear regression to the relationship between segment length and the corresponding average curve length.

Sevcik’s Algorithm (Sevcik 2006) is another method for estimating the fractal dimension of a time series. Sevcik’s Algorithm calculates the autocorrelation function of the time series and then uses a specialized algorithm to estimate the fractal dimension based on the decay rate of the autocorrelation.

Dimensional Complexity (DCx, Pritchard and Duke 1995) is a measure used to assess the complexity of a dynamical system, capturing the richness and diversity of its dynamics by evaluating the system’s behavior in its phase space. This is done by laying a grid over a phase space reconstructed from the time-series and estimating the amount of squares/cubes the trajectory of the signal in this space fills as it evolves through time.

Hurst Exponent (Hurst 1951) is a measure of long range temporal correlation (LRTC) of a time series. It describes the rate at which autocorrelations between value pairs in the time series decay as the time distance between the pair increases. The interpretation of the Hurst exponent in the context of complexity is not straightforward, as a value of H = 0.5 indicates no temporal correlations while values of 0 < H < 0.5 indicate the presence of anti-correlations, stronger when closer to 0 and values of 0.5 < H < 1 indicate the presence of correlations which are stronger when closer to 1. For monofractals, the Hurst exponent (H) directly relates to the fractal dimension (D) following the formula D = 2-H. Correspondingly, a smaller value of H entails a larger fractal dimension and vice versa.

Detrended Fluctuation Analysis (DFA, Peng et al. 1995) is a method for estimating the Hurst exponent of a time-series. DFA involves dividing the time series into smaller segments, removing the local trends within each segment, and then calculating the fluctuation of the detrended data as a function of segment size. By examining the relationship between the fluctuation and the segment size, DFA computes a scaling exponent, which is an estimation of the Hurst exponent.
