# Supplementary material for: Meditation and complexity: a review and synthesis of evidence
Source: Neurosci Conscious. 2025 May 28;2025(1):niaf013. doi: 10.1093/nc/niaf013 (PMC12118461; doi:10.1093/nc/niaf013)
Supplement: niaf013_Supp [file niaf013_supp.zip › suppl_data/Supplementary Figures 1-4.pdf]

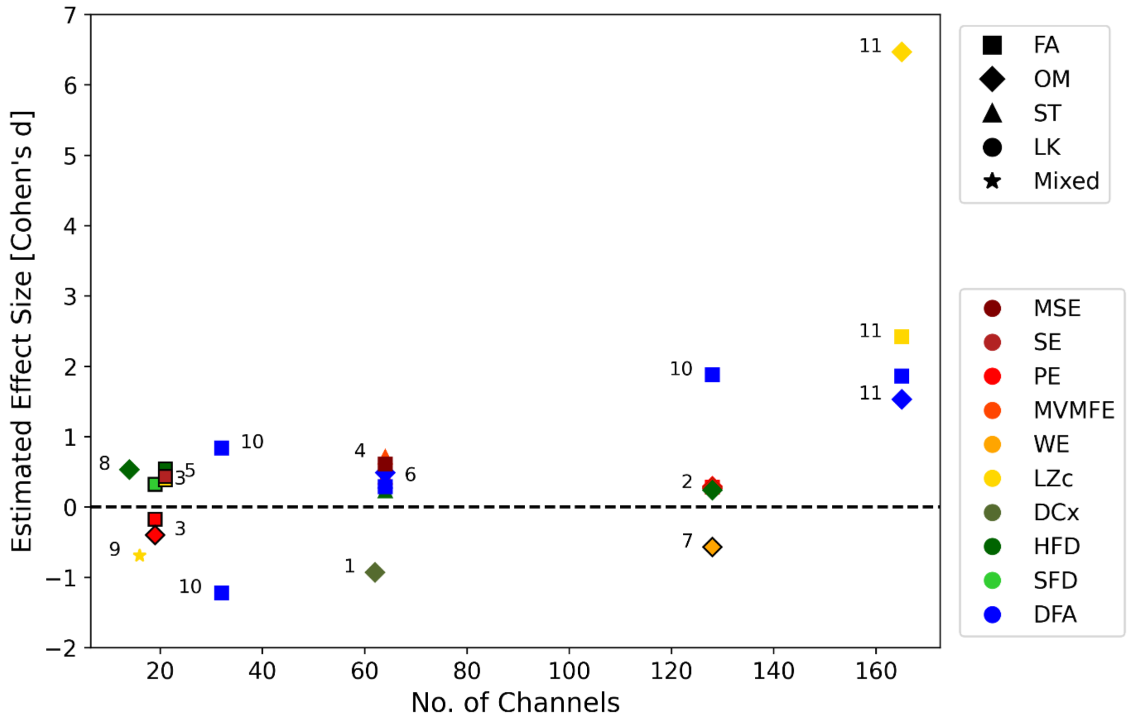

Supplementary Figure 1. A summary of the within-subject studies comparing meditative state to a control condition of rest or mind-wandering, showing estimated effect size as a function of the number of M/EEG channels used for data acquisition. Shapes indicate meditation style, colors indicate type of complexity measure.

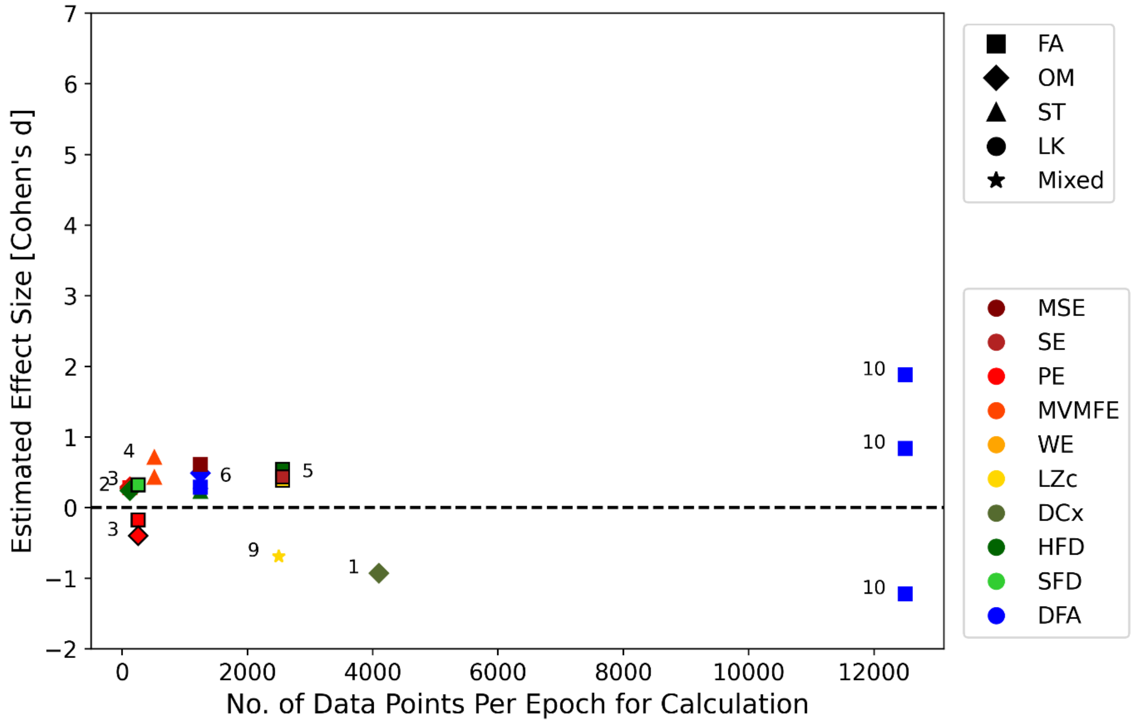

Supplementary Figure 2. A summary of the within-subject studies comparing meditative state to a control condition of rest or mind-wandering, showing estimated effect size as a function of the number of data points in each epoch used for measure calculation. Shapes indicate meditation style, colors indicate type of complexity measure. Note that some of the reviewed studies are omitted from this graph as they did not report information regarding number of data points or length of time-window per epoch

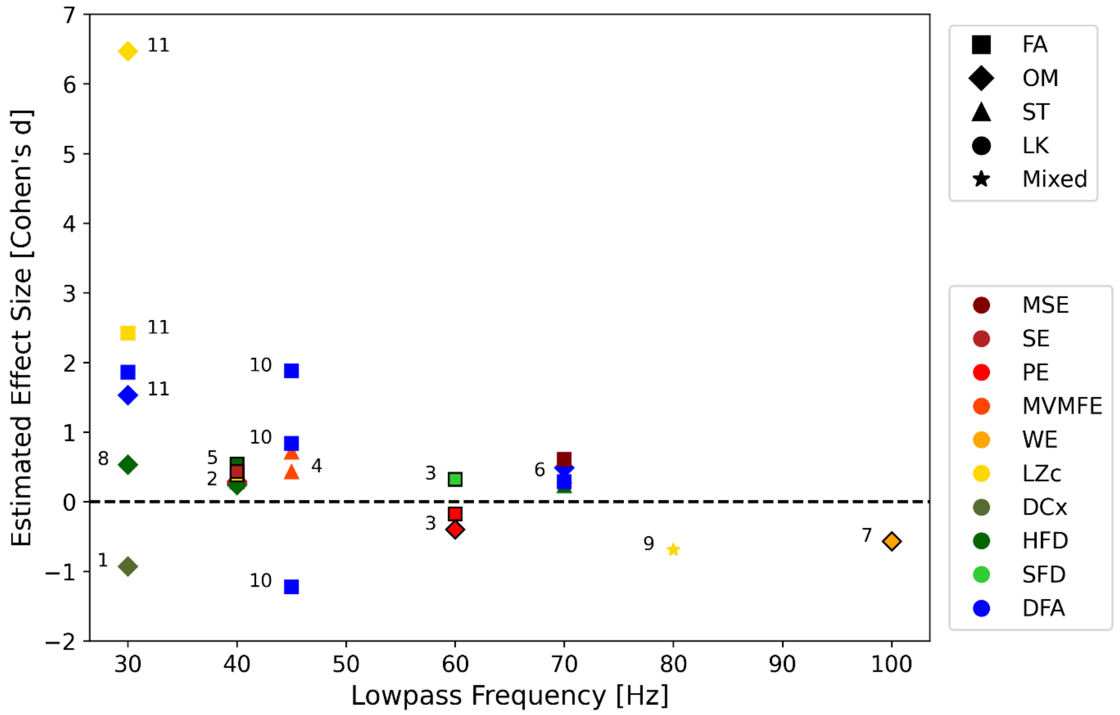

Supplementary Figure 3. A summary of the within-subject studies comparing meditative state to a control condition of rest or mind-wandering, showing estimated effect size as a function of the value of the low-pass filter applied prior to measure calculation. Shapes indicate meditation style, colors indicate type of complexity measure.

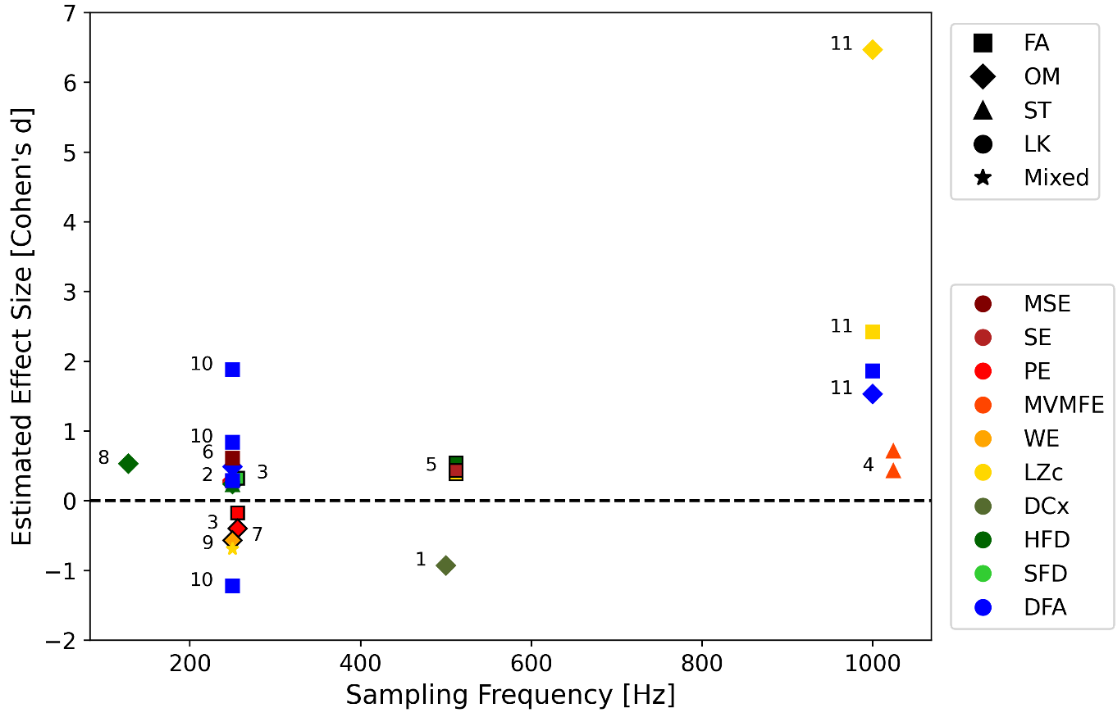

Supplementary Figure 4. A summary of the within-subject studies comparing meditative state to a control condition of rest or mind-wandering, showing estimated effect size as a function of sampling frequency. Shapes indicate meditation style, colors indicate type of complexity measure. Note that in some studies, data was down-sampled prior to measure calculation and that we denote here the down-sampled value.
